# FTO rs9939609 genotype and behavioral determinants of Mediterranean diet adherence in young women: exploratory insights into potential metabolic relevance

**DOI:** 10.3389/fnut.2026.1771565

**Published:** 2026-07-17

**Authors:** Małgorzata Obara-Gołębiowska, Katarzyna Eufemia Przybyłowicz, Anna Danielewicz, Tomasz Sawicki

**Affiliations:** 1 Department of Clinical, Developmental and Educational Psychology, Faculty of Social Sciences, University of Warmia and Mazury in Olsztyn, Olsztyn, Poland; 2 Department of Human Nutrition, Faculty of Food Sciences, University of Warmia and Mazury in Olsztyn, Olsztyn, Poland

**Keywords:** eating behavior, FTO rs9939609, functional foods, health education and health promotion, Mediterranean diet, metabolic health, nutrigenomics

## Abstract

**Introduction:**

Metabolic disorders such as obesity and insulin resistance result from complex interactions between genetic, behavioral, and dietary factors. The fat mass and obesity-associated (FTO) gene has been repeatedly linked to impaired appetite control and higher obesity risk, yet the moderating role of functional dietary patterns such as the Mediterranean diet remain unclear. Rich in bioactive compounds and metabolic benefits, this diet offers a model for studying gene–diet interactions in metabolic health.

**Objectives:**

This pilot investigation aimed to provide an integrative perspective linking the FTO rs9939609 genotype with eating-related emotions, habitual behavior, and adherence to a Mediterranean-style diet in women with different body weight profiles.

**Methods:**

This pilot study examined associations between the FTO rs9939609 genotype, eating behaviors, and adherence to the Mediterranean diet among 46 women aged 18–35 years (normal weight: *n* = 24; overweight or obesity: *n* = 22). Eating behaviors were assessed with the Questionnaire of Eating-Related Behaviors (QERB), and diet quality was evaluated using the Mediterranean Pyramid (MedPyr) index. Genotyping was performed by Sanger sequencing.

**Results:**

Women with overweight or obesity showed higher BMI (*p* < 0.001) and greater habitual overeating (*p* = 0.007), particularly among FTO AT/AA carriers with overweight or obesity. Total QERB scores were higher in both TT (*p* = 0.037) and AT/AA (*p* = 0.005) subgroups. No significant genotype- or BMI-related differences were observed in overall MedPyr adherence. However, AT/AA carriers with overweight or obesity reported more frequent vegetable intake (*p* = 0.037), while AT/AA controls consumed eggs more often (*p* = 0.015).

**Conclusion:**

Adherence to the Mediterranean diet, a functional dietary pattern rich in polyphenols, fiber, and unsaturated fats, may interact with genetic predispositions related to obesity risk. These exploratory findings suggest that dietary context may shape behavioral tendencies in FTO risk-allele carriers, although confirmation in larger samples is required.

## 1 Introduction

Obesity and related metabolic disorders are multifactorial conditions influenced by biological, psychological, and environmental determinants that jointly shape eating behavior, diet quality and body weight. Among these, dietary patterns, particularly functional diets rich in bioactive compounds, are recognized as key modulators of metabolic health and gene–environment interactions. Beyond lifestyle and socio-environmental factors, genetic predisposition plays a crucial role in obesity susceptibility, particularly variants of the fat mass and obesity-associated (FTO) gene (1–4). Carriers of the risk allele (A) of FTO rs9939609 typically exhibit greater energy intake, a stronger preference for calorie-dense foods, and heightened neural reactivity to food-related cues (5–8). The rs9939609 polymorphism consists of three genotypes: TT (wild-type homozygotes), AT (heterozygous carriers), and AA (homozygous risk-allele carriers).

However, growing evidence indicates that the relationship between FTO and obesity extends beyond purely metabolic mechanisms and involves behavioral regulation influencing both dietary choices and metabolic outcomes. Studies suggest that FTO-related risk may also manifest through behavioral and emotional pathways, including impulsivity, emotional vulnerability, and cognitive restraint. For instance, Harbron et al. (9) found that carriers of FTO risk alleles displayed greater hunger, emotional disinhibition, and higher intake of high-fat and refined carbohydrate foods, along with more depressive symptoms. Similarly, Abdella et al. (10) observed that although the AA/AT genotypes were associated with higher BMI, differences in eating behavior and food craving were mediated by cognitive restraint, particularly among younger individuals, suggesting adaptive self-regulation in response to genetic vulnerability. Consistent with these findings, A-allele carriers have been reported to consume more added sugars in healthy populations (11).

Research also highlights the modifying role of diet composition and lifestyle in how FTO influences body weight and overall metabolic health. In a randomized controlled trial, Huang et al. (12) showed that participants carrying the A allele experienced greater reductions in food cravings and appetite when following a high-protein, hypocaloric diet, indicating that macronutrient composition can modulate appetite-related genetic effects. Consistent with this, Przeliorz-Pyszczek and Regulska-Ilow (13) reviewed converging evidence suggesting that appropriate macronutrient balance may downregulate FTO expression and reduce obesity risk. These findings align with current perspectives emphasizing the role of functional foods and nutrient quality in metabolic regulation and obesity prevention. In contrast, large-scale population research, such as that of Johnson et al. (14), demonstrated that both FTO genotype and dietary energy density independently predicted fat mass in adolescents, without evidence for a gene–diet interaction, implying additive rather than synergistic effects. A recent meta-analysis further demonstrated that FTO variants may also influence responsiveness to diet and exercise interventions (15).

Conversely, recent cross-national data indicate that healthy dietary patterns may buffer genetic susceptibility. Livingstone et al. (16) reported that adherence to diets rich in vegetables, fruits, and whole grains attenuated the association between FTO rs9939609 and obesity across seven European cohorts. Similarly, adherence to a Mediterranean-style diet has been shown to mitigate genetic risk among European youth (17). This dietary pattern, characterized by high intake of polyphenols, unsaturated fatty acids, and dietary fiber, represents a functional nutrition model supporting metabolic balance. However, individuals with the AA genotype, typically associated with higher obesity risk, might paradoxically report healthier eating patterns, reflecting spontaneous or implicit behavioral adaptation rather than deliberate control (4, 10, 16). Likewise, lifestyle-related factors such as physical activity and cognitive restraint appear to moderate genotype effects. Taken together, these findings indicate that the influence of FTO is not deterministic but context-dependent, shaped by psychosocial, dietary, and behavioral factors.

Emerging evidence therefore supports the hypothesis that genetic susceptibility may be moderated by behavioral and self-regulatory factors that shape eating patterns and body weight (4, 10, 18). These mechanisms may also interact with diet composition and nutrient quality, influencing metabolic efficiency and adaptive energy balance. Dysregulated emotional responses, impulsivity, and maladaptive coping strategies have been consistently linked to emotional eating and loss of dietary restraint (19, 20). Such patterns suggest that genetic vulnerability may be amplified or mitigated by psychosocial and behavioral mechanisms. Although physical activity did not significantly moderate FTO-related outcomes in prior pilot data (21), both that study and other reports (20) indicate that lifestyle and adaptive eating behaviors may buffer the behavioral expression of genetic risk. This aligns with emerging perspectives in functional nutrition, suggesting that behavioral and nutritional self-regulation may partly offset genetic predispositions to metabolic dysregulation. Moreover, integrative frameworks in precision nutrition highlight how gene–nutrient and behavioral interactions jointly influence metabolic and emotional regulation in obesity (22).

Although FTO has been extensively studied in metabolic and neurobiological contexts, its role in shaping psychological correlates of eating behavior remains underexplored. Integrating psychological, nutritional, and genetic factors may thus enhance understanding of compensatory adaptations that support healthy metabolic function despite inherited risk. Understanding how genetic and emotional regulation mechanisms interact could illuminate why some individuals with genetic susceptibility maintain a healthy body weight despite elevated risk alleles.

Given the limited understanding of how genetic and psychological factors jointly shape dietary patterns, this pilot investigation offers an integrative perspective linking the FTO rs9939609 genotype with eating-related emotions, habitual behavior, and adherence to a Mediterranean-style dietary pattern in women with different body weight profiles (16, 17). Extending prior analyses conducted in this cohort that primarily focused on psychological and lifestyle correlates of body weight, the present study explicitly incorporates diet quality indicators, enabling a more comprehensive gene–behavior–diet framework. By emphasizing dietary adherence rather than body weight alone, the study contributes to the growing body of evidence on functional dietary patterns as potential behavioral contexts in which genetic susceptibility to obesity may be expressed or attenuated.

By combining genetic and behavioral indicators within the same cohort, the study aims to explore whether risk allele carriers display behavioral patterns suggestive of compensatory self-regulation or, conversely, persistent vulnerabilities related to overeating (10, 18). Such an approach may refine the conceptualization of obesity as a multifactorial phenomenon by linking psychological and nutritional determinants with genotype-related susceptibility within a biopsychosocial framework (4, 22).

Building on the psychological framework previously established in this cohort (21), the present pilot study adopts an exploratory design integrating the FTO rs9939609 genotype with indicators of emotional, habitual, and restrictive eating alongside measures of overall dietary composition. The primary objective was to examine BMI-related behavioral and dietary differences, whereas genotype-stratified analyses were treated as exploratory, given the modest subgroup sizes. By combining genetic, psychological, and dietary data, this work seeks to provide preliminary insights into multidimensional mechanisms linking genotype, eating behavior, and diet quality. These exploratory analyses are intended to inform future, adequately powered studies and the development of integrative biopsychosocial models of eating behavior and weight regulation (22). To our knowledge, few pilot studies have jointly examined the FTO rs9939609 genotype, detailed eating-behavior profiles, and Mediterranean diet adherence within the same cohort of young adult women.

## 2 Materials and methods

### 2.1 Study design, participants and sample size calculation

Details on the study design and methods are available elsewhere (21). Briefly, this exploratory pilot study was conducted on a cohort of 46 young adult women aged 18–35 years recruited between March and September 2020 from the University of Warmia and Mazury in Olsztyn, Poland, through social media and local announcements. Participants were assigned to one of two groups based on body mass index (BMI): a normal-weight control group and a group of women with overweight or obesity after meeting inclusion criteria. All participants were in good general health, had no history of eating disorders, psychiatric diagnoses, or chronic metabolic diseases, and provided written informed consent prior to participation. The study was approved by the Ethics Committee for Scientific Research of the University of Warmia and Mazury in Olsztyn, Poland (approval no. 3/2019, approval date: 21 October 2019), and conducted in accordance with the Declaration of Helsinki. Participants were recruited using convenience sampling; therefore, the potential for selection bias and limited generalizability should be considered when interpreting the findings.

All assessments were conducted during a single laboratory session. After providing informed consent, participants completed the Questionnaire of Eating-Related Behaviors (QERB) and dietary interview, followed by anthropometric measurements and buccal cell collection. Testing took place in the morning, in fasting conditions, and lasted approximately 45 min per participant.

Initial sample size calculation was performed and presented in previous work (21) which showed that 62 women (31 in each group) should be included in the study. That calculation assumed 30% difference in QERB score between women with normal weight and overweight and obesity. However, due to the fact that a different endpoint was chosen in this study, it was decided to perform a *post hoc* sample size calculation. Assuming alpha 0.05 and power 80% using the mean and SD of Mediterranean Pyramid score (MedPyr) among TT carriers in the control group and assuming that MedPyr score for A-allele carriers is 10 and 15% lower, the expected sample size should be 148 (37 vs.111) and 64 (16 vs.48) individuals, respectively [when enrolment ratio is 3:1 for women with overweight and obesity vs. the control group (based on FTO allele variants distribution in the studied group) or 110 and 50, respectively (with equal enrolment ratio)].

Given that the final analytical sample was smaller than the estimated sample sizes required for adequately powered comparisons, the present analyses were interpreted as exploratory and hypothesis-generating.

### 2.2 Anthropometric assessment

Body weight and height were measured using a calibrated medical scale and stadiometer (SECA 515mBCA, Allers, Hamburg, Germany and Radwag WPT 100/200, Poland, respectively), with participants wearing light clothing and no shoes. Body mass index (BMI) was calculated as weight (kg) divided by height squared (m^2^). BMI over 25 kg/m^2^ and 30 kg/m^2^ were classified as overweight and obesity, respectively. BMI was used as the criterion for selecting the study group—women with overweight and obesity (O/O groups) – and the control group – women with normal body weight (C).

### 2.3 Eating behavior assessment

Self-regulation of eating in terms of emotional and habitual overeating and dietary restrictions was assessed using the QERB developed by Ogińska-Bulik (23). The questionnaire measures eating-related patterns and allows the diagnosis of tendencies toward overeating, prediction of weight gain risk, and identification of the causes of excessive food intake leading to overweight or obesity.

Each affirmative answer was scored with one point, except for five reverse-coded items where one point was awarded for a “no” response. The total score ranges from 0 to 30, while each subscale (habitual overeating, emotional overeating, dietary restrictions) ranges from 0 to 10. Higher scores indicate more dysfunctional eating behaviors, which may be associated with a greater risk of developing disordered eating or excessive body weight (10, 19). Reliability coefficients for the QERB reported in the original validation study were as follows: α = 0.671 for the total score, α = 0.632 for habitual overeating, α = 0.560 for emotional overeating, and α = 0.560 for dietary restrictions (23). Eating behaviors assessed using the QERB were analyzed as three subscales: Emotional Overeating (EO), Habitual Overeating (HO), and Dietary Restrictions (DR).

### 2.4 Nutritional assessment

Participants were asked to answer the self-administered, semi-quantitative food frequency questionnaire (FFQ) comprising 72 food items, measuring consumption of specific foods over the previous year. The reproducibility and relative validity of the questionnaire was assessed and published (24). To assess adherence to healthy eating guidelines, we chose the Mediterranean diet model based on of the Mediterranean pyramid (MedPyr) recommendations (25). The calculations of the score follows the algorithm developed by Tong et al. (26). The method includes 15 food groups classified as those to be consumed in high, moderate, or low quantities and continuous scoring ranging from 0 to 1 is assigned according to the degree of adherence to the recommendation. Vegetables, legumes, and fish are recommended for consumption in high quantities, scores increase proportionally from 0 (no consumption) to 1 (recommended level). For foods recommended in low quantities such as red meat, processed meat, potatoes, and sweets, scoring is reversed. Fruits, nuts, cereals, dairy products, white meat, eggs, and alcohol in recommended to consume in moderation, a score of 1 is given for intake within the recommended range, 0 for no intake, and 0.5 when consumption exceeds the midpoint of the recommendation by twofold. For alcohol, moderate consumption receives 1 point, abstinence 0.5 points, and excessive intake 0 points. Olive oil was scored as 1 if was the main source of fat (26, 27).

### 2.5 Genotyping of FTO rs9939609

Buccal epithelial cell samples were collected from each participant using sterile swabs. The collected material was sent to a certified external laboratory (Genomed S. A., Warsaw, Poland) for genetic analysis. Genotyping of the FTO rs9939609 (T/A) polymorphism was performed according to the laboratory’s internal procedures. PCR products were purified using the Exo-SAP protocol to remove unincorporated primers and dNTPs. Sequencing reactions were prepared using BigDye™ Terminator v3.1 chemistry together with BigDye™ Terminator v1.1/v3.1 5 × sequencing buffer, appropriate primers (5 pmol), and 25–50 ng of DNA template, and were carried out under the cycling conditions provided by the manufacturer (96 °C for 1 min, followed by 25 cycles at 96 °C for 10 s, 54 °C for 5 s, and 60 °C for 4 min). Purified sequencing products were separated by capillary electrophoresis on a 3730xl DNA Analyzer (Thermo Fisher Scientific). Genotypes were classified as AA (homozygous risk allele), AT (heterozygous), or TT (homozygous wild-type) based on the nucleotide identified at the rs9939609 polymorphic site in the sequencing trace, according to the laboratory’s internal interpretation protocol. All genotype distributions met the expected Hardy–Weinberg equilibrium (1, 3, 4). Observed genotype frequencies in our sample were consistent with distributions previously reported for European populations for the rs9939609 variant (28, 29).

### 2.6 Statistical analysis

The normality of the variables was examined using the Shapiro–Wilk test. Continuous variables are presented as the median and interquartile range (IQR). Differences between the control and overweight or obesity groups and between FTO rs9939609 genotypes were analyzed using the Mann–Whitney U test. Due to limited subgroup sizes and to increase the statistical power of the analyses, the dominant genetic model (AA + AT vs. TT) was applied. This approach is commonly used in exploratory genetic analyses with limited sample sizes to improve statistical stability. Spearman’s rank correlation test was used to assess relationships between the frequency of food consumption and QERB results across FTO genotypes in both groups.

Associations between the Mediterranean Pyramid (MedPyr) score and QERB results were evaluated using standard logistic regression models. Odds ratios (ORs) and 95% confidence intervals (CIs) were estimated within the logistic regression framework. Model parameters were obtained using the default numerical optimization procedure (Quasi-Newton method) implemented in the statistical software, and their significance was assessed using the χ^2^ Wald test. MedPyr scores were categorized into approximate tertiles, with the lowest tertile serving as the reference group (OR = 1). QERB scores were modeled as continuous variables, and the obtained ORs represent a one-point change in QERB. Two models were fitted: Crude (unadjusted) and Model 1 (adjusted for BMI, kg/m^2^). Linear trends were assessed by assigning median values within each MedPyr tertile and modeling these values as continuous predictors. All tests were two-tailed, and *p* values below 0.05 were considered statistically significant. Statistical analyses were performed using TIBCO® Statistica™ version 13.3 (TIBCO Software Inc., Tulsa, OK, United States).

## 3 Results

### 3.1 Group characteristics and food intake patterns

Before pooling AT and AA genotypes for statistical analyses, the full genotype distribution was examined. Primary group comparisons were conducted by BMI category, whereas genotype-stratified analyses were treated as exploratory due to limited subgroup sizes. In the C group (*n* = 24), the frequencies were TT = 7 (29.2%), AT = 10 (41.7%), and AA = 7 (29.2%). In the O/O group (n = 22), the distribution was TT = 8 (36.4%), AT = 10 (45.5%), and AA = 4 (18.2%). Overall, the frequencies in the total sample (*N* = 46) were TT = 15 (32.6%), AT = 20 (43.5%), and AA = 11 (23.9%).

Table 1 presents the distribution of anthropometric characteristics, food intake frequencies, and QERB scores across BMI groups and FTO genotypes. As expected, BMI was markedly higher in participants with overweight or obesity (O/O) than in controls (*p* = 0.001 for TT; *p* < 0.001 for AT/AA), but did not differ within the O/O or control groups between FTO rs9939609 genotype variants. Regarding dietary intake, O/O participants carrying the AT/AA genotype reported more frequent vegetable consumption than their control counterparts (*p* = 0.037), whereas AT/AA participants in the control group reported more frequent egg consumption than TT controls (*p* = 0.015). Intake frequencies of other food items did not differ significantly among subgroups.

**Table 1:** Differences of food intake frequencies, MedPyr score and QERB results among FTO gene variants.

Characteristics	Control		*P*	Overweight/obesity		*P*	TT *P*_*C* vs. *O*_	AT/AA *P*_*C* vs. *O*_	TT*_C_* vs. AT/AA*_O_ P*
	FTO			FTO					
	TT	AT/AA		TT	AT/AA				
*n*	7	17		8	14				
Age, years	21.0 (1.0)	21 (2.0)	0.737	20.5 (8.0)	20.5 (6.5)	0.808	1.000	0.501	0.849
BMI, kg/m^2^	21.71 (2.78)	20.33 (3.02)	0.066	29.29 (7.92)	30.73 (9.98)	0.973	0.001	<0.001	<0.001
Food intake									
Vegetables [times/d]	2.12 (1.40)	2.05 (0.63)	0.568	1.25 (1.45)	2.3 (2.63)	0.142	0.772	0.037	0.126
Legumes [times/wk]	0.79 (1.29)	0.58 (0.37)	0.089	0.61 (1.08)	1.14 (2.08)	0.973	0.594	0.719	0.449
Fruits [times/d]	1.67 (2.08)	2.11 (2.08)	0.611	2.17 (2.45)	1.55 (4.59)	0.918	0.862	0.953	0.970
Nuts [times/d]	0.9 (1.08)	0.91 (1.02)	0.657	1.19 (1.10)	0.75 (2.75)	0.973	0.862	0.766	0.794
Cereals [times/d]	2.08 (2.28)	1.86 (2.21)	1.000	1.59 (1.20)	1.80 (0.72)	0.306	0.524	0.225	0.263
Dairy [times/d]	1.99 (1.00)	2.34 (1.42)	0.899	1.91 (1.74)	2.12 (2.65)	0.474	0.954	0.648	0.576
Fish [times/wk]	1.16 (1.50)	1.00 (2.58)	0.391	0.71 (0.74)	1.16 (1.03)	0.121	0.771	0.188	0.056
Red meat [times/wk]	1.71 (2.16)	2.29 (1.45)	0.750	1.37 (1.16)	0.90 (1.56)	0.374	0.117	0.474	0.278
Processed meat [times/wk]	4.79 (6.49)	5.79 (4.00)	0.657	4.66 (6.57)	5.18 (9.93)	0.973	0.772	0.751	0.576
White meat [times/wk]	1.50 (1.51)	1.50 (0.92)	0.362	0.58 (0.92)	1.04 (2.43)	0.771	0.857	0.228	0.937
Egg [times/wk]	2.08 (2.21)	5.00 (2.92)	0.015	2.35 (4.21)	1.89 (5.79)	0.973	0.295	0.952	0.215
Potatoes [times/wk]	2.29 (3.29)	3.58 (2.21)	0.949	3.93 (3.84)	2.29 (2.67)	0.656	0.816	0.873	0.623
Sweets [times/wk]	8.41 (6.20)	12.27 (8.80)	0.341	5.94 (9.56)	6.78 (9.43)	0.657	0.385	0.648	0.192
Alcohol [units/d]	1.05 (1.05)	1.57 (2.66)	0.228	1.27 (1.41)	1.15 (3.77)	0.838	0.603	0.843	0.279
MedPyr score [points]	8.56 (2.05)	10.00 (2.19)	0.374	8.13 (3.06)	8.18 (2.71)	0.865	0.183	0.226	0.068
QERB									
Emotional overeating [points]	3 (3)	4 (2)	0.847	5 (4)	5.5 (5)	0.864	0.162	0.034	0.093
Habitual overeating [points]	3 (3)	3 (1)	0.648	6 (3)	6.5 (2.5)	0.467	0.007	0.007	0.005
Dietary Restrictions [points]	3 (3)	4 (5)	0.949	5 (3)	4 (4)	0.627	0.557	0.100	0.196
Total [points]	10 (8)	10 (6)	0.949	17 (6)	16 (5)	0.918	0.037	0.005	0.012

Data are presented as median and interquartile range (IQR). BMI, body mass index; C, control group; O, overweight/obesity; d, day; wk, week; QERB, Questionnaire of Eating-Related Behaviors; MedPyr, Mediterranean Pyramid index. Genotypes were analyzed as TT vs. AT/AA (AA and AT combined due to limited subgroup sizes). *P* values were obtained using the Mann–Whitney U test and considered significant if *p* < 0.05.

Accordingly, the MedPyr score showed no significant variation between genotypes or BMI groups, although slightly higher values were observed among control AT/AA carriers (10.0 vs. 8.6 points). A non-significant trend was observed when comparing control TT carriers and O/O AT/AA carriers (8.56 [2.05] vs. 8.18 [2.71], *p* = 0.068).

Analysis of QERB scores showed no differences between AT/AA and TT carriers within either the O/O or control groups. However, when genotypes were analyzed separately, several domains were higher in the O/O group compared with controls. Among AT/AA carriers, the O/O group showed significantly higher emotional overeating and habitual overeating (*p* = 0.034 and *p* = 0.007, respectively) as well as total QERB scores (*p* = 0.005). Among TT carriers, participants in the O/O group had higher habitual overeating (*p* = 0.007) and total QERB scores (*p* = 0.037). Additionally, significantly higher habitual overeating (6.5; IQR: 2.5 vs. 3.0; IQR: 3.0, *p* = 0.005) and total QERB scores (16.0, IQR: 5.0 vs. 10.0; IQR: 8.0, *p* = 0.012) were observed for O/O AT/AA carriers compared with control TT carriers.

Analyses comparing individual genotype variants for anthropometric and QERB outcomes were reported previously. Detailed analyses of food intake and MedPyr scores are presented in Supplementary Table S1. No differences were observed within the O/O or control groups between AA and AT variants. Similarly, neither AT nor AA carriers differed in food consumption or MedPyr score when compared with TT carriers (data not shown).

Given the pilot and hypothesis-generating character of the study, *post hoc* power was estimated. Power for the comparison between TT and AT/AA carriers for BMI was 2.5% in the O/O group and 59.6% in the control group; for MedPyr it was 3.5% in the O/O group and 6.0% in the control group; and for QERB it was 3.6% in the O/O group and 5.9% in the control group. The power for the difference between the control and O/O groups among AT/AA carriers was 28.8% for the MedPyr score and 80.9% for QERB.

### 3.2 Correlation analysis

**Figure 1:**
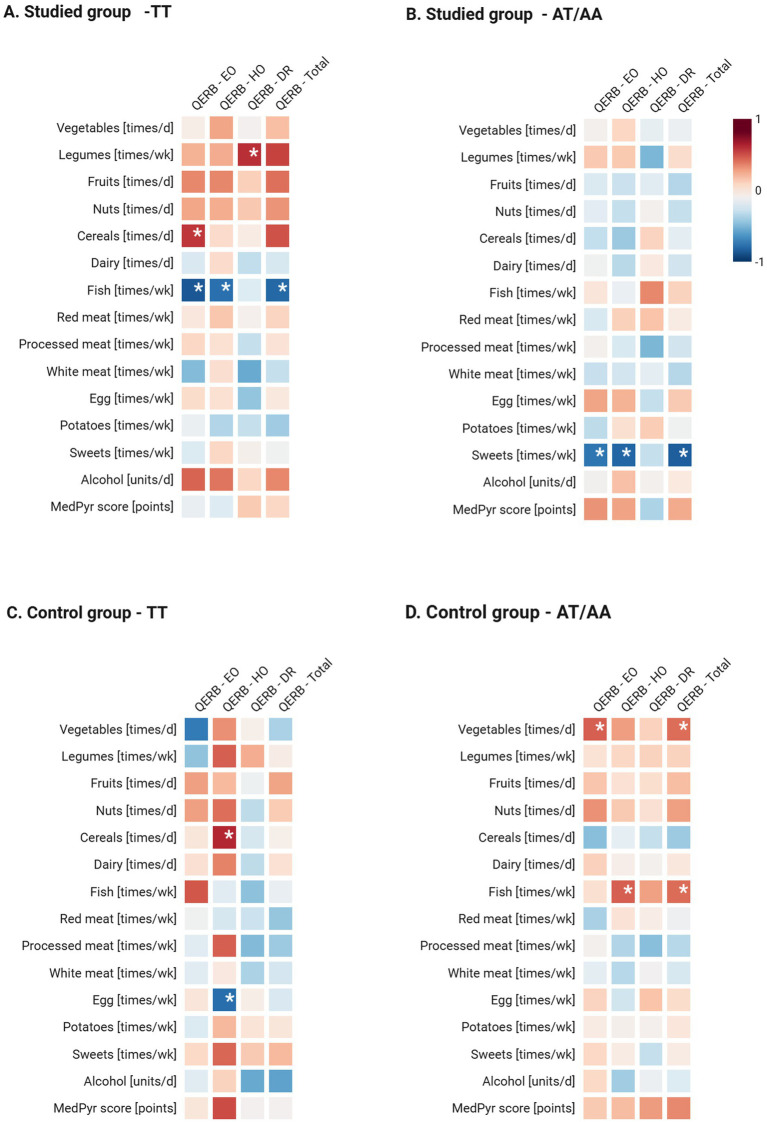
Heatmaps of correlations between frequencies of food intake, MedPyr score, and QERB subscales across groups stratified by weight status and FTO rs9939609 genotype: **(A)** participants with overweight or obesity who were TT carriers; **(B)** participants with overweight or obesity who were AT/AA carriers; **(C)** controls who were TT carriers; **(D)** controls who were AT/AA carriers. Blue shades indicate positive correlations and red shades indicate negative correlations according to Spearman’s r (*p* < 0.05 considered statistically significant). Created in BioRender. Sawicki, T. (2026) https://BioRender.com/sd7cbma.

Figure 1 presents heatmaps illustrating correlations between food intake frequency and eating behavior subscales across study groups stratified by FTO rs9939609 genotype and weight status. In the O/O group with the TT genotype (panel A), positive associations were observed for legume consumption and dietary restriction (*r* = 0.745) and for cereal consumption and emotional overeating (*r* = 0.723), whereas fish intake correlated negatively with emotional (*r* = −0.854) and habitual (*r* = −0.753) overeating and total QERB score (*r* = −0.787).

Among O/O AT/AA carriers (panel B), sweets consumption was inversely correlated with emotional (*r* = −0.727) and habitual (*r* = −0.793) overeating and total QERB score (*r* = −0.827). In the control TT group (panel C), habitual overeating was positively correlated with cereal intake frequency (*r* = 0.777) and negatively correlated with egg intake frequency (*r* = −0.762). In the control group with AT/AA genotypes (panel D), vegetable intake frequency correlated positively with emotional overeating (*r* = 0.601) and total QERB score (*r* = 0.559), whereas fish intake frequency correlated positively with habitual overeating (*r* = 0.601) and total QERB score (*r* = 0.565).

### 3.3 Associations between MedPyr score and QERB results

The relationships between adherence to the Mediterranean diet (MedPyr) and eating behavior patterns (QERB) across FTO genotypes are summarized in Table 2. Among individuals with the TT genotype, no significant associations were observed between the MedPyr score and any QERB subscales, including emotional overeating, habitual overeating, dietary restriction, or total QERB score. Similarly, in participants carrying the AT/AA genotype, no statistically significant relationships were detected. Adjustment for BMI (Model 1) did not alter these findings.

**Table 2:** MedPyr score in relation to QERB results among different FTO variants.

Variable	MedPyr score			*P*_trend_
	Bottom tertile <8.014	Middle tertile 8.014–9.324	Upper tertile > 9.324	
FTO = TT				
*n*				
Emotional overeating				
Crude	1.00	1.05 (0.64, 1.71)	0.85 (0.48, 1.50)	0.408
Model 1	1.00	1.02 (0.60, 1.74)	0.79 (0.37, 1.73)	0.304
Habitual overeating				
Crude	1.00	0.93 (0.50, 1.73)	0.75 (0.40, 1.42)	0.079
Model 1	1.00	0.48 (0.09, 2.57)	0.69 (0.30, 1.55)	0.676
Dietary restriction				
Crude	1.00	1.28 (0.54, 3.01)	0.98 (0.56, 1.73)	0.882
Model 1	1.00	1.24 (0.49, 3.16)	1.01 (0.53, 1.91)	0.944
Total QERB				
Crude	1.00	1.03 (0.78, 1.35)	0.93 (0.73, 1.18)	0.442
Model 1	1.00	0.99 (0.68, 1.43)	0.90 (0.65, 1.24)	0.196
FTO = AT/AA				
*n*				
Emotional overeating				
Crude	1.00	0.99 (0.67, 1.48)	1.29 (0.86, 1.93)	0.272
Model 1	1.00	0.97 (0.63, 1.52)	1.31 (0.86, 1.98)	0.304
Habitual overeating				
Crude	1.00	1.22 (0.77, 1.93)	1.46 (0.95, 2.25)	0.064
Model 1	1.00	1.30 (0.72, 2.35)	1.56 (0.95, 2.54)	0.106
Dietary restriction				
Crude	1.00	1.05 (0.71, 1.56)	1.17 (0.79, 1.74)	0.069
Model 1	1.00	1.04 (0.62, 1.75)	1.18 (0.78, 1.79)	0.118
Total QERB				
Crude	1.00	1.04 (0.87, 1.23)	1.16 (0.96, 1.40)	0.099
Model 1	1.00	1.05 (0.82, 1.34)	1.19 (0.96, 1.47)	0.090

Data are presented as odds ratio (OR) and 95% confidence interval (95% CI). Reference group: participants from the bottom MedPyr tertile. Crude – unadjusted; Model 1 – adjusted for BMI (kg/m^2^). QERB, Questionnaire of Eating-Related Behaviors; MedPyr, Mediterranean Pyramid index.

However, non-significant trend-level patterns were observed in AT/AA carriers, where higher MedPyr adherence was accompanied by tendencies toward higher habitual overeating (*p* = 0.064 in the crude model), dietary restriction (*p* = 0.069 in the crude model), and total QERB scores (*p* = 0.099 in the crude model; *p* = 0.090 in the adjusted model), suggesting a genotype-dependent direction of association. Given the number of statistical comparisons performed, the results should be interpreted cautiously. None of the observed associations remained statistically significant after correction for multiple testing.

## 4 Discussion

### 4.1 Overview of main findings

This pilot exploratory study examined relationships between the FTO rs9939609 genotype, eating behavior patterns, and dietary habits among women with normal weight and those with overweight or obesity. Although no significant genotype- or BMI-related differences were found in overall adherence to the Mediterranean diet (MedPyr) or alcohol intake, several subtle genotype-specific tendencies emerged. Women with overweight or obesity carrying the AT/AA genotype reported more frequent vegetable consumption compared with their normal-weight counterparts, whereas within the control group AT/AA carriers reported more frequent egg consumption than TT participants. Associations between eating behavior and diet quality also varied by genotype. In AT/AA carriers, higher MedPyr adherence co-occurred with trends toward greater habitual overeating and higher total QERB scores, whereas in TT carriers higher intake of vegetables and fish was associated with lower emotional and habitual overeating. This pattern may tentatively suggest that genetic predisposition interacts with nutritional context in shaping eating-behavior tendencies, possibly reflecting adaptive or compensatory behavioral self-regulation processes. Given the small and underpowered sample size, these tendencies should be interpreted cautiously, particularly in light of the limited precision of the estimates. Accordingly, the present findings should be interpreted primarily as hypothesis-generating signals rather than confirmatory evidence. Nonetheless, the findings tentatively suggest that FTO-related behavioral patterns may not be uniformly maladaptive and may differ depending on genotype and dietary environment (21, 30).

### 4.2 Genetic and behavioral mechanisms

Variants of the FTO gene are among the most consistently replicated genetic correlates of obesity, with the A allele of rs9939609 associated with higher BMI, increased energy intake, and preferential responses to energy-dense foods (1, 2, 5). Neuroimaging findings by Melhorn et al. (6), showing altered corticolimbic activation among A-allele carriers, and the reward responsivity model proposed by Stice and Yokum (31) further support a genotype-linked obesity-vulnerability phenotype. However, these mechanisms are not deterministic. Behavioral and psychological factors, including dietary restraint, impulsivity, and emotion regulation, can moderate how genetic susceptibility manifests in eating behaviors (3, 18).

The present findings extend earlier analyses from the same cohort (21) by integrating detailed dietary indicators derived from the FFQ and MedPyr scoring. Because participants were unaware of their FTO status, the observed associations likely reflect spontaneous rather than intentional adaptations. This interpretation aligns with theoretical accounts suggesting that genetic influences on eating behavior may be mediated by automatic cognitive–emotional processes involved in reward and satiety regulation (3, 6, 31).

Population-level data further support the context-dependent role of FTO. Livingstone et al. (16) showed that diet quality is inversely associated with adiposity independent of genotype, while Johnson et al. (14) observed primarily additive effects of rs9939609 and dietary energy density on fat mass. Other studies have reported that higher protein or fiber intake may mitigate appetite-related or metabolic correlates among A-allele carriers (12, 13). A recent study further demonstrated that eating-behavior traits such as restraint, disinhibition, and food cravings may shift rapidly following dietary changes (32). Collectively, these findings suggest that the impact of FTO may depend more on the surrounding behavioral environment than on genotype alone.

### 4.3 Potential compensatory patterns in A-allele carriers

The present pilot study found that among AT/AA carriers, higher adherence to Mediterranean-style dietary recommendations coexisted with trends toward greater habitual overeating and higher total QERB scores. Rather than indicating inconsistent reporting, this pattern may reflect potential compensatory behavioral strategies, whereby individuals predisposed to dysregulated eating increase engagement with structured or health-oriented dietary habits. This interpretation is consistent with findings showing higher cognitive restraint among physically active AA carriers (33) and with evidence that cognitive control can buffer the relationship between BMI and food cravings (10). Although the study by Abdella et al. (10) included both men and women, the underlying mechanism remains relevant for interpreting behavioral patterns observed in the present female sample. By contrast, treatment-seeking adults with the A allele have demonstrated greater disinhibition and internal hunger cues in less supportive environments (9), illustrating the moderating role of psychological and situational context.

In the present study, TT carriers demonstrated negative associations between healthy food intake and maladaptive eating tendencies, suggesting a more stable regulatory profile. Such patterns may indicate genotype-specific adaptive responses; however, confirmation in larger and longitudinal studies is required (15). Recent findings from Anguah et al. (32) demonstrate that changes in eating-behavior traits can occur rapidly in response to dietary interventions, further supporting the conceptualization of self-regulatory mechanisms as dynamic and context-dependent. Intervention and cohort studies further suggest that dietary composition may modulate FTO-related appetite and metabolic responses (12, 13), while large population studies typically report independent effects of diet quality and FTO variants on obesity risk (14, 16). The co-occurrence of greater habitual overeating with higher dietary restriction observed in AT/AA carriers may reflect increased cognitive effort rather than effective regulatory control (8, 11). Further complexity is suggested by several unexpected food-specific associations observed in the present study. An additional finding that merits consideration concerns the strong inverse associations between sweets consumption and emotional overeating, habitual overeating, and total QERB scores observed among women with overweight or obesity carrying the AT/AA genotype. At first glance, this pattern appears counterintuitive, as previous research has generally linked FTO risk alleles with stronger preferences for energy-dense foods and heightened reward responsiveness (5, 8). Several explanations may be considered. First, sweets consumption in this subgroup may reflect controlled or planned inclusion of palatable foods within an otherwise structured dietary pattern rather than impulsive or emotionally driven eating. Second, moderate consumption of preferred foods may reduce feelings of deprivation and psychological tension around eating, thereby lowering the tendency toward emotional or habitual overeating. Such an interpretation would be consistent with models suggesting that excessive restriction may paradoxically contribute to overeating episodes (10, 32). Third, and perhaps most importantly, given the small subgroup size (*n* = 14), these correlations may simply reflect sampling variability or the influence of a limited number of participants and should therefore be interpreted with considerable caution until replicated in larger samples.

Similarly, the positive associations observed between vegetable consumption and maladaptive eating indicators, as well as between fish intake and habitual overeating among control participants carrying the AT/AA genotype, were not anticipated. One possible explanation is that individuals reporting greater overeating tendencies may simultaneously engage in compensatory efforts to improve diet quality by increasing the consumption of foods generally perceived as healthy. Such patterns may reflect attempts at dietary self-regulation rather than direct causal relationships between specific foods and eating behaviors. Nevertheless, the possibility that these findings reflect random variation related to multiple testing and limited statistical power cannot be excluded. Taken together, these findings highlight the complex interplay between genetic predisposition and cognitive-emotional regulation of eating behavior (22).

### 4.4 Practical implications and biopsychosocial integration

While speculative, such perspectives align with emerging precision nutrition approaches. Despite its exploratory nature, this study contributes to understanding how genetic susceptibility may interact with psychological and behavioral factors in shaping eating patterns and potential metabolic risk (4, 24). Integrating genotyping with eating-behavior and diet-quality measures provides an initial empirical step toward identifying behavioral profiles that may be relevant for individualized dietary strategies. These findings also illustrate how self-regulatory mechanisms, such as cognitive restraint or habitual overeating, may operate differently across FTO genotypes, particularly when embedded within specific dietary contexts.

Within a biopsychosocial framework previously described by Obara-Gołębiowska et al. (21), the present results suggest that personalized approaches combining psychological and nutritional components may potentially be useful for individuals carrying genetic risk alleles. Interventions such as cognitive-behavioral therapy, structured dietary self-monitoring, or targeted improvements in diet quality could, in theory, support adaptive regulatory processes among genetically vulnerable individuals (4, 22). However, such implications remain preliminary and should be confirmed in controlled intervention studies.

### 4.5 Limitations and future directions

Several limitations must be acknowledged. First, this was a small-sample pilot exploratory study, which substantially limits statistical power and generalizability. The limited number of TT carriers further reduced the precision of subgroup analyses. The structure of the dataset did not meet key assumptions required for more precise analyses, mainly due to the imbalance between genotype frequencies and markedly unequal group sizes. Moreover, the categorical nature of the primary outcomes and the low prevalence of certain genotypes further restricted the feasibility of reliably modeling genotype and weight-status interactions. To minimize sex-related heterogeneity in dietary behaviors, eating regulation, and genotype–behavior interactions, the authors focused on a female subsample. However, the inclusion of only women limits the generalizability of the findings and precludes the assessment of potential sex-dependent genotype effects. Future studies should therefore include both women and men to determine whether the observed genotype–behavior associations differ across sexes. Therefore, all findings should be interpreted with particular caution given the exploratory scope of the present pilot study and the modest statistical power of several subgroup analyses. Particular caution is warranted with respect to nominal *p*-values and the width of the corresponding confidence intervals, as both type I and type II errors cannot be excluded. In addition, associations approaching conventional levels of statistical significance (e.g., *p* values between 0.05 and 0.10) should not be interpreted as evidence of biological or behavioral effects but rather as preliminary signals requiring replication in larger, adequately powered samples. In addition, logistic regression models based on MedPyr tertiles should be interpreted cautiously, as the small sample size and subgroup structure may have resulted in model instability. Furthermore, the use of a dominant genetic model, although justified by subgroup sizes, may have obscured potential differences between heterozygous and homozygous carriers. Future studies with larger samples should therefore analyze all genotype variants separately.

Second, the cross-sectional design precludes causal inference, and the use of self-reported food-frequency data introduces potential recall and reporting bias. Moreover, although trends were observed, none of the associations survived correction for multiple testing. Additionally, although the applied questionnaires were designed to assess relatively stable and habitual behaviors over extended periods of time, hormonal status and menstrual-cycle phase were not assessed. These factors may influence appetite regulation, food preferences, reward sensitivity, and emotional eating behaviors and could therefore contribute to variability in the studied outcomes. Future studies should incorporate detailed assessments of hormonal status and menstrual-cycle phase to improve the precision and interpretability of genotype–behavior associations.

Thus, given the limited sample size and the novelty of combining genetic and behavioral perspectives, this investigation was intended to be hypothesis-generating rather than confirmatory and was designed to explore emerging genotype–behavior patterns that may inform future large-scale, theory-driven studies (15). It is also worth noting that QERB, as a self-report measure, may not fully capture contextual or physiological determinants of eating behavior. In addition, the modest internal consistency of some QERB subscales may have introduced measurement error and attenuated the strength of observed associations. Unmeasured psychosocial variables, such as impulsivity, stress reactivity, or executive control, may also contribute to the observed patterns (18, 19). Future studies should involve larger, demographically diverse cohorts and adopt longitudinal or experimental designs to determine whether the observed genotype-specific associations reflect genuine behavioral adaptation, contextual moderation, or random variation. Integrating nutritional biomarkers, metabolic parameters, and molecular indicators of gene expression would further enhance understanding of gene–diet–behavior mechanisms. Such integrative approaches would allow more precise modeling of how cognitive–emotional regulation interacts with metabolic predisposition (4, 22).

## 5 Conclusion

This pilot exploratory study provides preliminary evidence that the relationship between FTO rs9939609 and eating behavior may involve subtle behavioral modulation shaped by dietary context. Women carrying the risk allele displayed a mixed pattern of heightened overeating tendencies alongside stronger dietary restriction, which may reflect potentially compensatory or effortful regulation rather than genuinely adaptive control. These findings should be regarded as preliminary and hypothesis-generating. They do not establish causal or mechanistic effects but instead highlight potential directions for future integrative research.

From a nutrition science standpoint, these results tentatively support the concept that balanced dietary patterns, such as Mediterranean-style diets, may interact with behavioral regulation to potentially buffer genetic susceptibility to metabolic dysregulation. Within a biopsychosocial framework, they underscore the importance of viewing obesity not as a purely metabolic condition but as a dynamic interplay among genetic, cognitive, and behavioral processes (4, 22). Replication in larger, longitudinal samples will be essential to determine whether the associations observed here reflect compensatory adaptation, contextual moderation, or random variation.

## Data Availability

The original contributions presented in the study are publicly available. This data can be found here: https://doi.org/10.5281/zenodo.21261206.
