# Supplementary material for: FTO rs9939609 genotype and behavioral determinants of Mediterranean diet adherence in young women: exploratory insights into potential metabolic relevance
Source: Front Nutr. 2026 Jul 17;13:1771565. doi: 10.3389/fnut.2026.1771565 (PMC13423642; doi:10.3389/fnut.2026.1771565)
Supplement: Supplementary file 1 [file Table_1.DOCX]

Supplementary Material

FTO rs9939609 Genotype and Behavioral Determinants of Mediterranean Diet Adherence: Implications for Metabolic Health in Young Women

Małgorzata Obara-Gołębiowska¹, Katarzyna Eufemia Przybyłowicz², Anna Danielewicz² and Tomasz Sawicki² *

^1^ Department of Clinical, Developmental and Educational Psychology, Faculty of Social Sciences, University of Warmia and Mazury in Olsztyn, Olsztyn, Poland

^2^ Department of Human Nutrition, Faculty of Food Sciences, University of Warmia and Mazury in Olsztyn, Olsztyn, Poland,

***** Correspondence: m.obara-golebiowska@uwm.edu.pl

**Table S1.** Differences of food intake frequencies, MedPyr score and QERB results among AA and AT variants of FTO gene.

| **Food intake Characteristics** | **Control** | | ***P*** | **Overweight/Obesity** | | ***P*** | **AA**  ***P_C vs. O_*** | **AT**  ***P_C vs. O_*** |
| --- | --- | --- | --- | --- | --- | --- | --- | --- |
|  | **FTO** | |  | **FTO** | |  |  |  |
|  | **AA** | **AT** |  | **AA** | **AT** |  |  |  |
| *n* | 7 | 10 |  | 4 | 10 |  |  |  |
| Vegetables [times/d] | 1.97 (1.08) | 2.5 (1.97) | 0.961 | 2.01 (2.36) | 1.23 (0.74) | 0.289 | 0.637 | 0.076 |
| Legumes [times/wk] | 0.79 (1.29) | 0.9 (1.5) | 0.922 | 1.6 (6.1) | 0.42 (0.58) | 0.222 | 0.567 | 0.492 |
| Fruits [times/d] | 1.67 (2.67) | 1.77 (2.15) | 0.807 | 2.86 (2.53) | 1.81 (2.12) | 0.724 | 0.777 | 0.910 |
| Nuts [times/d] | 1.34 (0.96) | 0.83 (1.23) | 0.464 | 1.3 (1.04) | 1.11 (0.99) | 0.944 | 0.925 | 0.970 |
| Cereals [times/d] | 2.5 (1.97) | 1.94 (2.38) | 0.435 | 1.69 (1.37) | 1.5 (1.2) | 0.944 | 0.299 | 0.705 |
| Dairy [times/d] | 2.05 (0.96) | 1.84 (1.54) | 0.696 | 1.77 (1.18) | 2 (1.74) | 0.832 | 0.395 | 0.970 |
| Fish [times/wk] | 1.5 (1.13) | 0.58 (3.16) | 0.696 | 0.69 (0.4) | 0.71 (0.74) | 0.616 | 0.085 | 0.732 |
| Red meat [times/wk] | 1.95 (2.16) | 1.54 (2.42) | 0.732 | 1.37 (0.93) | 1.27 (1.32) | 0.831 | 0.448 | 0.880 |
| Processed meat [times/wk] | 3.5 (3.9) | 7.04 (7.62) | 0.262 | 7.97 (5.32) | 3.63 (4.12) | 0.357 | 0.156 | 0.241 |
| White meat [times/wk] | 1.5 (1.51) | 1.5 (0.92) | 0.374 | 1.04 (1.67) | 0.58 (0.92) | 0.703 | 0.487 | 0.427 |
| Egg [times/wk] | 2.08 (2.92) | 1.62 (2.21) | 0.343 | 1.89 (4.39) | 2.35 (4.21) | 0.943 | 0.775 | 0.701 |
| Potatoes [times/wk] | 2.29 (4.09) | 3.29 (3.84) | 0.731 | 4.76 (4.12) | 3.93 (2.75) | 0.522 | 0.704 | 0.879 |
| Sweets [times/wk] | 6.0 (8.17) | 9.94 (10.24) | 0.262 | 8.87 (11.41) | 5.94 (8.56) | 0.832 | 0.637 | 0.241 |
| Alcohol [units/d] | 1.44 (1.99) | 0.87 (0.98) | 0.526 | 4.14 (7.46) | 1.17 (1.01) | 0.077 | 0.299 | 0.970 |
| MedPyr score [points] | 9.02 (2.62) | 8.19 (2.7) | 0.306 | 10.29 (3.61) | 8.01 (2.04) | 0.138 | 0.777 | 0.427 |

Data are presented as median and interquartile range (IQR). BMI – body mass index; C – control group; O – overweight/obesity; d – day; wk – week; MedPyr – Mediterranean Pyramid index. P values were obtained using the Mann–Whitney U test and considered significant if p < 0.05.
